# Adenovirus Type 4 Respiratory Infections among Civilian Adults, Northeastern United States, 2011–2015^1^

**DOI:** 10.3201/eid2402.171407

**Published:** 2018-02

**Authors:** Adriana E. Kajon, Daryl M. Lamson, Camden R. Bair, Xiaoyan Lu, Marie L. Landry, Marilyn Menegus, Dean D. Erdman, Kirsten St. George

**Affiliations:** Lovelace Respiratory Research Institute, Albuquerque, New Mexico, USA (A.E. Kajon, C.R. Bair);; New York State Department of Health, Albany, New York, USA (D.M. Lamson, K. St. George);; Centers for Disease Control and Prevention, Atlanta, Georgia, USA (X. Lu, D.D. Erdman);; Yale University School of Medicine, New Haven, Connecticut, USA (M.L. Landry);; University of Rochester Medical Center, Rochester, New York, USA (M. Menegus)

**Keywords:** adenovirus type 4, ARD, acute respiratory disease, ILI, influenza-like illness, next-generation sequencing, genome typing, viruses, respiratory infections, United States, adenovirus, HAdV-4, outbreak, civilians

## Abstract

Human adenovirus type 4 (HAdV-4) is most commonly isolated in military settings. We conducted detailed molecular characterization on 36 HAdV-4 isolates recovered from civilian adults with acute respiratory disease (ARD) in the northeastern United States during 2011–2015. Specimens came from college students, residents of long-term care facilities or nursing homes, a cancer patient, and young adults without co-morbidities. HAdV-4 genome types 4a1 and 4a2, the variants most frequently detected among US military recruits in basic training before the restoration of vaccination protocols, were isolated in most cases. Two novel a-like variants were recovered from students enrolled at a college in Tompkins County, New York, USA, and a prototype-like variant distinguishable from the vaccine strain was isolated from an 18-year-old woman visiting a physician’s office in Ulster County, New York, USA, with symptoms of influenza-like illness. Our data suggest that HAdV-4 might be an underestimated causative agent of ARD among civilian adults.

Human adenovirus type 4 (HAdV-4), the only human adenovirus classified within species E, was first identified in the early 1950s in association with military outbreaks of febrile respiratory illness and is well-recognized worldwide as a prevalent causative agent of acute respiratory disease (ARD) and ocular disease (*1*–*6*). Surveillance studies conducted in the United States and other countries have demonstrated a leading role for this particular adenovirus type in the etiology of outbreaks of febrile respiratory illness in military recruit training facilities (*7*–*11*), where crowding and environmental contamination appear to facilitate transmission among nonvaccinated trainees (*12*,*13*). By using restriction enzyme analyses of viral DNA, several studies have reported extensive intratypic genetic variability for HAdV-4 (*14*–*16*). Two major clusters of genetic homology have been identified among circulating genomic variants: prototype (p)–like viruses, which are closely related to prototype strain RI-67, and a-like viruses, which exhibit, among other characteristics, distinct *Bam*HI restriction profiles (*15*), a distinct inverted terminal repeat (*17*,*18*), and a different genetic make-up in the E3 region (A.E. Kajon, unpub. data).

HAdV-4 respiratory infections are preventable by vaccination with the live oral formulation of the nonattenuated p-like strain exclusively licensed for military use (*19*,*20*). After 15 years of discontinuation of HAdV-4 vaccination protocols with the consequent resurgence of continuous outbreaks of HAdV-4–associated illness in US recruit training facilities nationwide, US Department of Defense reinstated the vaccine in November 2011, dramatically reducing the number of cases of HAdV infection in basic training camps (*21*,*22*).

The absence of a sentinel system for HAdV surveillance outside of the military has made assessing the burden of disease attributable to HAdV-4 infection among civilians difficult. The limited epidemiologic data available in the published literature suggest that respiratory disease associated with HAdV-4 infection is detected at a significantly lower frequency than disease associated with species C or B HAdV types among children and that HAdV-4 infection occurs rarely among civilian adults (*23*–*27*).

Consequently, the apparent increased frequency of detection of cases and case clusters of HAdV-4 respiratory infection in the northeastern United States, documented by the New York State Department of Health (NYSDOH) or the Centers for Disease Control and Prevention (CDC) (data not shown), caught our attention. In this article, we report the molecular characterization of 36 select HAdV-4 isolates from a selection of retrospectively evaluated cases among civilians.

## Methods

### Source of Specimens

We obtained HAdV-4–positive specimens from patients with ARD/influenza-like illness (ILI) characterized by fever >37.8°C and cough, sore throat, or other respiratory symptoms. We selected cases that were originally identified by the NYSDOH as part of its activities for the US Sentinel Physician ILI Surveillance Network (https://www.cdc.gov/flu/weekly/pdf/flu-surveillance-overview) or that represented special HAdV cases referred to the CDC for investigation because of their clinical disease severity or occurrence during an outbreak. Institutional review board review was not required for the processing of clinical samples or for the evaluation of patient information, which were obtained during routine diagnostic workups at the Clinical Virology Laboratory, Yale–New Haven Hospital (New Haven, Connecticut, USA), and the University of Rochester Medical Center (Rochester, New York, USA). Review board approval was also not required for the typing protocol used on deidentified HAdV isolates at the Lovelace Respiratory Research Institute (Albuquerque, New Mexico, USA).

### Virus Isolation and Initial Identification

Virus isolation from respiratory specimens and typing was performed at CDC (Atlanta, Georgia, USA) or the Virology Laboratory, Wadsworth Center NYSDOH (Albany, New York, USA). The virus isolates were initially identified as HAdV-4 by molecular procedures as previously described (*28*,*29*).

### Genome Typing by Restriction Enzyme Analysis

Cultured isolates were shipped to Lovelace Respiratory Research Institute for propagation and extraction of intracellular viral DNA from infected A549 cell monolayers as previously reported (*30*). We digested viral DNA samples with a panel of 6 endonucleases to identify genomic variants following the initial guidelines of Li and Wadell (*15*) and as previously applied to the genomic characterization of US military strains (*16*) to facilitate comparisons. In brief, we digested 1 μg of purified viral DNA with *Bam*HI, *Dra*I, *Eco*RI, *Eco*RV, *Sma*I, and *Xho*I (New England Biolabs, Ipswich, MA, USA) in a 20-μL reaction following the manufacturer’s recommended conditions. For the isolates selected for complete genomic sequencing, we performed genome typing by in silico digestion of their viral DNA with the same panel of enzymes using Geneious Pro 9 (Biomatters Ltd, Auckland, New Zealand) (*31*).

### Genome Sequencing and Analysis

We then carried out next-generation sequencing reactions at the Wadsworth Center’s Applied Genomics Technology Core with the purified viral genomic DNA prepared at Lovelace Respiratory Research Institute. The 12 isolates that were selected for next-generation sequencing were representative of the set of identified variants on the basis of restriction enzyme analysis, location, and date of detection. We prepared libraries with the NexteraXT kit (Illumina, San Diego, CA, USA) and performed paired-end sequencing on the Illumina MiSeq using the NextSeq 500 cycle v2 kit (Illumina). We assembled all genomic sequences de novo using the Illumina BaseSpace cloud application for SPAdes 3.5 (http://spades.bioinf.spbau.ru/release3.5.0/manual.html); we then remapped the sample sequences to the consensus sequence using Geneious Pro 9 (https://www.geneious.com/). Confirmation of sequence accuracy for specific regions of the genome was carried out by Sanger sequencing of PCR amplicons as needed. We annotated the HAdV-4 genomes using curated reference sequences available for the prototype RI-67 strain (GenBank accession no. AY594253), the vaccine strain CL68578 (GenBank accession no. AY487947), and several military isolates representing previously described genomic variants (GenBank accession nos. EF371058, AY599837, and AY599835).

For phylogenetic analysis, we aligned the genomic sequences generated in this study with reference strains (Table 1) using MAFFT in Geneious Pro 9. We constructed a maximum-likelihood tree on the basis of the Kimura 2-parameter model (*32*) with 500 bootstrap replicates using MEGA6 (*33*).

**Table 1:** Clinical isolates and reference strains used in the phylogenetic analysis of HAdV-4 strains recovered from cases of acute respiratory infection detected in northeastern United States, 2011–2015*

Virus name	Place and year of isolation	Genome type	GenBank accession no.
Isolates from this study			
TB071911	Yale, CT, 2011	4a2	KY996453
12-12752 (NY7)	NY, 2012	4a2	KY996450
12-27440 (NY8)	NY, 2012	4a1	KY996451
13-5497 (NY11)	NY, 2013	4a *Sma*I v	KY996449
14-4876 (NY16)	NY, 2014	4a2	KY996448
14-9111 (NY17)	NY, 2014	4a1	KY996442
14-33430 (NY20)	NY, 2014	4a1	KY996445
14-38662 (NY21)	NY, 2014	4a1	KY996443
14-38813 (NY22)	NY, 2014	4a1	KY996444
15-418 (NY23)	NY, 2015	4a1	MF002042
15-3477 (NY24)	NY, 2015	4a *Sma*I/*Xho*I v	KY996446
15-4054 (NY25)	NY, 2015	4p	KY996447
Reference strains			
RI-67	Fort Leonard Wood, MO, 1952	4p	AY594253
CL68578†	Camp Lejeune, NC, 1965	4p	AY487947
RU-2533	Cape May, NJ, 1966	4p	MF002043
NHRC90339	Cape May, NJ, 2001	4p4	EF371058
NHRC42606	Fort Jackson, SC, 2002	4a2	AY599835
NHRC3	Brooks Air Force Base, TX, 2003	4a1	AY599837

*HAdV-4, human adenovirus type 4; NHRC, Naval Health Research Center; v, variant. †Vaccine strain.

## Results

### Case Descriptions

The HAdV-4–positive ARD/ILI cases we evaluated occurred in otherwise healthy teenagers, young adults in college, and older adult patients who were residing in long-term care facilities, with 1 older adult patient hospitalized in a cancer center. Some cases required prolonged hospitalization or had fatal outcomes.

#### Nosocomial Outbreak of HAdV-4 Respiratory Infection in Long-Term Care Facility for Elderly—Boston, Massachusetts, April–May 2006

A detailed description of this outbreak was published by Kandel et al. in 2010 (*34*). In brief, the outbreak occurred in a unit with 40 residents of mean age 88 (range 66–99) years. During April–May 2006, fifteen residents had symptoms of ARD. HAdV-4 infection was confirmed for 4 residents who had positive virus culture results through PCR amplification and sequencing of the hexon hypervariable regions 1–6 as described by Lu and Erdman (*28*). The nasopharyngeal aspirates from 3 symptomatic residents gave negative virus culture results, and the remaining 8 residents were not sampled. Three of the 4 patients with confirmed HAdV-4 infections died of complications from ARD. Isolates from 2 of the confirmed cases of HAdV-4–associated pneumonia identified during this outbreak were processed for viral DNA extraction and detailed characterization. The 2 respiratory isolates were genome typed as variant 4a1 by restriction enzyme analysis at Lovelace Respiratory Research Institute.

#### Adult Case of Severe Pneumonia—Connecticut, July 2011

One author (M.L.L.) was involved in testing, consulting, and advising on this case as the clinical laboratory director and as an infectious disease specialist. A 26-year-old man with an unremarkable medical history sought treatment at an emergency department for a 3-day illness involving severe headache, photophobia, nausea, vomiting, and chills. He had seen his doctor 2 days earlier and was treated with azithromycin without improvement. His lumbar puncture results were normal, but a chest radiograph showed a left upper lobe infiltrate. Blood work showed a normal white blood cell count with 27% band cells, anemia, thrombocytopenia, and elevated creatinine. The patient was admitted and treated with ceftriaxone and azithromycin. On the following day, he experienced severe respiratory distress, so he was intubated and transferred to intensive care. His nasopharyngeal swab tested positive by panadenovirus PCR at the Clinical Virology Laboratory, Yale New Haven Hospital. On the 5th day after hospital admission, the patient remained febrile, and his chest radiograph showed diffuse bilateral infiltrates; dialysis was initiated for a creatinine of 4.9 mg/dL (or 430 µmol/L, reference range 53–106 µmol/L). By using the pan-adenovirus PCR, the plasma viral load was determined to be 1.16 × 10^4^ copies/mL. Molecular typing conducted at CDC confirmed the presence of HAdV-4 in both the nasopharyngeal swab and plasma specimens. By using a HAdV-4–specific PCR (*35*), the plasma viral load was determined to be 5.00 × 10^5^ copies/mL. The patient eventually recovered with supportive therapy. The HAdV-4 isolate obtained from the nasopharyngeal swab was genome typed at Lovelace Respiratory Research Institute as variant 4a2 by restriction enzyme analysis.

#### Cases of ILI among College Students—New York, 2011–2015

**Figure 1:**
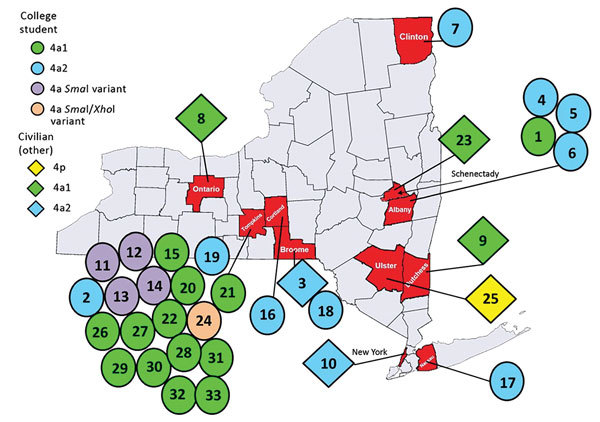
Geographic distribution of cases of human adenovirus type 4 (HAdV-4) infection identified by the New York State Department of Health through sentinel surveillance efforts targeting influenza-like illness (ILI), by HAdV-4 type, by type of civilian, by county, New York, USA, 2011–2015. Respiratory specimens were collected from patients with ILI at physicians’ offices, long-term care facilities, hospitals, and colleges and submitted to the Clinical Virology Laboratory at Wadsworth Center (Albany, New York, USA) to identify the causative agent.

During December 2011–October 2015, several HAdV-4–positive cases were identified at the Wadsworth Center Virology Laboratory among students enrolled at 7 colleges in 6 New York counties (Table 2; Figure 1). Persons arrived at their corresponding student health clinics with symptoms of ILI but tested negative for influenza by the CDC human influenza virus real-time reverse transcriptase PCR diagnostic panel. Four different HAdV-4 genomic variants were isolated from this group of patients 18–25 years of age (Table 2).

**Table 2:** Basic demographics, clinical characteristics, and virology findings of 33 cases of HAdV-4 acute respiratory infection detected by New York State Department of Health surveillance, New York, USA, 2011–2015*

Case ID	Specimen collection date	Specimen	Patient age, y/sex	Setting	County	Diagnosis	Genome type†
NY1	2011 Dec	NPS, OPS	19/M	College 1	Albany	ILI	4a1
NY2	2011 Dec	NSW	18/M	College 2	Tompkins	ILI	4a2
NY3	2012 Jan	NPS, OPS	29/M	Outpatient visit	Broome	ILI	4a2
NY4	2012 Jan	NPS, OPS	21/F	College 1	Albany	ILI	4a2
NY5	2012 Jan	NPS, OPS	21/M	College 1	Albany	ILI	4a2
NY6	2012 Jan	NPS, OPS	22/M	College 1	Albany	ILI	4a2
NY7	2012 Apr	NPS	22/F	College 3	Clinton	ILI	4a2‡
NY8	2012 Aug	NPS/TA	43/F	ICU	Ontario	Pneumonia, acute respiratory distress syndrome	4a1‡
NY9	2012 Sep	NPS	98/F	Nursing home	Dutchess	Pneumonia	4a1
NY10	2012 Oct	TS	43/M	Cancer center	New York	Fatal outcome	4a2
NY11	2013 Feb	NPS	21/M	College 4	Tompkins	ILI	4a *Sma*I v‡
NY12	2013 Feb	NPS, OPS	20/F	College 4	Tompkins	ILI	4a *Sma*I v
NY13	2013 Mar	NPS	19/M	College 4	Tompkins	ILI	4a *Sma*I v
NY14	2013 Apr	NPS, OPS	18/M	College 4	Tompkins	ILI	4a *Sma*I v
NY15	2013 Dec	NPS	21/F	College 4	Tompkins	ILI	4a1
NY16	2014 Feb	NPS	19/M	College 5	Cortland	ILI	4a2‡
NY17	2014 Mar	NPS	20/F	College 6	Nassau	ILI	4a2‡
NY18	2014 Mar	NPS	18/F	College 7	Broome	ILI	4a2
NY19	2014 May	NPS, OPS	19/F	College 4	Tompkins	ILI	4a2
NY20	2014 Oct	NPS	18/M	College 2	Tompkins	ILI	4a1‡
NY21	2014 Dec	NPS, OPS	25/F	College 4	Tompkins	ILI	4a1‡
NY22	2014 Dec	NPS	18/M	College 2	Tompkins	ILI	4a1‡
NY23	2015 Jan	NPS	13/M	Outpatient visit	Schenectady	ILI	4a1‡
NY24	2015 Feb	NPS, OPS	20/M	College 4	Tompkins	ILI	4a *Sma*I/*Xho*I v‡
NY25	2015 Feb	NPS, OPS	18/F	Outpatient visit	Ulster	ILI	4p‡
NY26	2015 Oct	NPS	21/M	College 2	Tompkins	ILI	4a1
NY27	2015 Oct	NPS	20/M	College 2	Tompkins	ILI	4a1
NY28	2015 Oct	NPS	18/F	College 2	Tompkins	ILI	4a1
NY29	2015 Oct	NPS	20/M	College 2	Tompkins	ILI	4a1
NY30	2015 Oct	NPS	18/F	College 2	Tompkins	ILI	4a1
NY31	2015 Oct	NPS	18/M	College 2	Tompkins	ILI	4a1
NY32	2015 Oct	NPS	19/F	College 2	Tompkins	ILI	4a1
NY33	2015 Oct	NPS	22/M	College 2	Tompkins	ILI	4a1

*HAdV-4, human adenovirus type 4; ICU, intensive care unit; ID, identification; ILI, influenza-like illness; NPS, nasopharyngeal swab; OPS, oropharyngeal swab; TA, tracheal aspirate; TS, throat swab; v, variant exhibiting unpublished profiles for the specified endonucleases. †Determined by restriction enzyme analysis of viral genomic DNA performed in vitro, in silico, or both with *Bam*HI, *Dra*I, *Eco*RI, *Eco*RV, *Sma*I, and *Xho*I and designated according to Li and Waddell (*15*). ‡Restriction enzyme analysis by in silico digestion.

#### Additional Cases of HAdV-4 Infection Identified by ILI Surveillance—New York, 2011–2015

During 2011–2015, ILI surveillance efforts identified 5 additional cases of acute HAdV-4 respiratory infection of variable severity (Table 2; Figure 1). These cases included infections in 2 adult patients (NY3 and NY25) sampled in physicians’ offices, a case of pneumonia reported in a nursing home (NY9), a fatal case involving respiratory complications in a patient at a cancer center (NY10), and a case of ARD detected in a teenager at a pediatric clinic (NY23).

#### Adult Case of Severe Pneumonia—Ontario County, New York, August 2012

One author (M.M.) was involved in testing, consulting, and advising on this case as the director of the Virology Laboratory at the Strong Memorial Hospital, University of Rochester Medical Center. A 43-year-old woman with cough and an unremarkable medical history sought treatment at the emergency department of University of Rochester Medical Center, Monroe County (NY8; Table 2; Figure 1). She was prescribed levofloxacin for presumed community-acquired pneumonia and sent home. Four days later, she was admitted with worsening cough, shortness of breath, and rigors and was found to have bilateral infiltrates on chest radiograph, anemia, and leukocytosis. She subsequently required intubation for declining respiratory status. Despite treatment with multiple broad-spectrum antimicrobial drugs, she experienced severe hypoxic hypercarbic respiratory failure requiring venous-venous extracorporeal mechanical oxygenation. Her nasopharyngeal swab was positive for HAdV by FilmArray (BioFire Diagnostics, bioMérieux, Marcy l’Etoile, France). She was treated with 3 doses of cidofovir. Her hospital course was complicated by severe acute kidney injury, acute tubular necrosis, and anuria requiring continuous veno-venous hemofiltration. Additional complications included cerebral edema, intracranial hemorrhage, and persistent hypertension. She was weaned from the ventilator after 40 days and was discharged to in-patient rehabilitation on day 53 of hospitalization. A year after discharge, she continued to experience bronchiectasis and dyspnea on exertion but had otherwise returned to her previous level of function. HAdV was isolated from cultures of the patient’s tracheal aspirate and nasopharyngeal swab, and a PCR of her peripheral blood demonstrated a virus load of 3.47 × 10^5^ copies/mL. Molecular typing at the Wadsworth Center identified the virus as HAdV-4. The HAdV-4 isolate was genome typed as variant 4a1 by both in vitro and in silico restriction enzyme analyses (Table 2).

### Virology Findings

**Figure 2:**
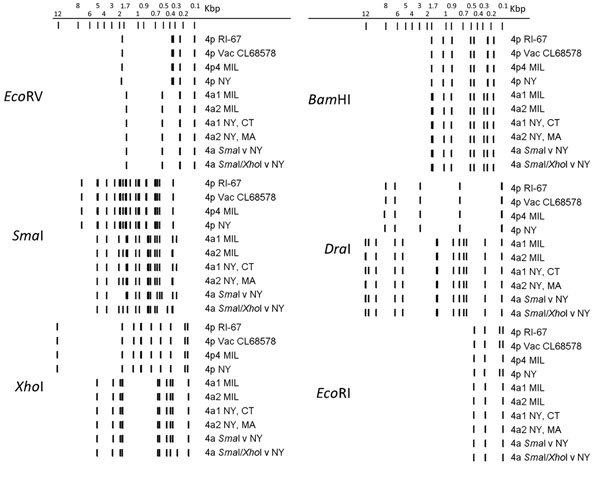
In silico restriction enzyme analysis of human adenovirus type 4 genomes representing the spectrum of genetic variability of the 36 isolates characterized in study of acute respiratory infection detected in the northeastern United States, 2011–2015. We generated restriction enzyme profiles for the completely sequenced genomes obtained in this study and from reference sequences available in GenBank using Geneious Pro (*31*). 4p4 MIL is isolate NHR90339, 4a1 MIL is isolate NHRC3, and 4a2 MIL is isolate 42606; 4a *Sma*I v is isolate NY11 (GenBank accession no. KY996449) and 4a *Sma*I/*Xho*I v is isolate NY24 (GenBank accession no. KY996446). MIL, military isolate; v, variant; Vac, vaccine strain.

**Figure 3:**
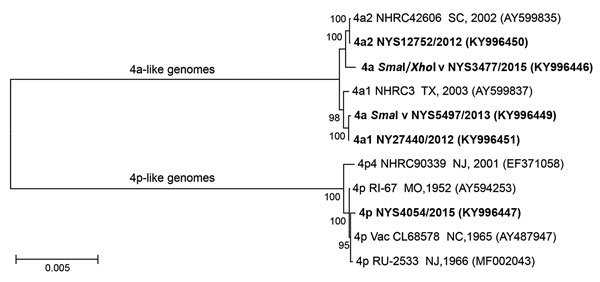
Phylogenetic analysis of complete genomic sequences of human adenovirus type 4 reference strains and clinical isolates representative of those examined in study of cases of acute respiratory infection detected in northeastern United States, 2011–2015. We inferred the phylogenetic tree using the maximum-likelihood method on the basis of the Kimura 2-parameter model (*32*). Evolutionary analyses were conducted in MEGA6 (*33*). Isolates sequenced in this study are in bold. GenBank accession numbers are in parentheses. Scale bar indicates substitutions per site.

Among these 36 select cases (some associated with outbreaks and some epidemiologically unlinked ARD cases) that occurred during 2009–2015 in the northeastern United States, 5 different genomic variants of HAdV-4 were identified by gel-based or in silico restriction enzyme analysis (Figure 2). Isolate NY25 (GenBank accession no. KY996447) was identified as 4p-like (Figure 3) and was indistinguishable from the prototype strain RI-67 or the vaccine strain CL68578 by restriction enzyme analysis (Figure 2). Of the 35 a-like isolates, 18 were classified as genome type 4a1, 12 as genome type 4a2, 4 as genome type 4a *Sma*I v (having a 4a-like genome with a novel *Sma*I profile), and 1 as genome type 4a *Sma*I/*Xho*I v (having a 4a-like genome with novel *Sma*I and *Xho*I profiles) (Table 2).

To investigate a possible transmission event of the vaccine strain administered orally to US military recruits since October 2011 (*22*), we further characterized the 4p-like strain isolated from case NY25 by whole-genome sequencing. We identified 10 point mutations scattered throughout the genome (6 nonsynonymous and a 3-nt [CAG] in-frame insertion at position 23402 within the L4 coding region and open reading frame of the 100-kDa protein), distinguishing the 2015 isolate from the vaccine strain, CL68578. Phylogenetic analysis of HAdV-4 genomic sequences (Figure 3) also showed this isolate to be more closely related to strains RI-67 and CL68578 than to strain NHRC 90339 (GenBank accession no. EF371058), which was isolated at the Coast Guard Recruit Training Center (Cape May, New Jersey, USA) in 2001 and is representative of genome type 4p4, the only p-like variant detected at military training facilities through 2011 (*16*) (A.E. Kajon, unpub. data). The phylogenetic analysis revealed 2 major clades, recapitulating the original observations and genomic clustering of variants by Li and Wadell (*15*). The analysis also showed the *Sma*I variant to be closely related to genome type 4a1 and the *Sma*I/*Xho*I variant to be closely related to genome type 4a2.

## Discussion

Enhanced influenza surveillance by public health laboratories initiated after the emergence of pandemic influenza A(H1N1) in 2009, as well as the wider availability of molecular diagnostic assays for multiple viral pathogens, have resulted in increased diagnostic efforts to determine the etiology of influenza-negative ILI, with consequent increased detection of HAdV-associated ARD. As part of our ongoing collaborative efforts to describe the molecular epidemiology and determine the prevalence of HAdV-associated respiratory disease, we examined HAdV-4 isolates recovered from college students with acute febrile respiratory illness in New York and several adults with severe respiratory disease in other locations in the northeastern United States. Restriction enzyme analysis with enzymes previously used to characterize HAdV strains from military recruits (*16*) and complete genomic sequencing identified 5 different genomic variants among the characterized clinical HAdV-4 isolates. Two of these variants, 4a1 and 4a2, had been previously identified in association with outbreaks of febrile respiratory illness in military recruit training facilities in the United States and found to be highly prevalent in the basic training environment nationwide before reinstatement of recruit vaccination protocols in 2011 (*16*) (A.E. Kajon, unpub. data). The genomic variant 4a1 was isolated from the respiratory specimens of 18 of 36 civilians and 4a2 from the respiratory specimens of 12 of 36 civilians retrospectively examined in this study. Two previously unreported variants closely related to 4a1 (*Sma*I v, n = 4) and 4a2 (*Sma*I/*Xho*I v, n = 1) were identified among the 8 examined cases detected at college 4 in Tompkins County, New York. Surprisingly, a p-like, vaccine-like strain was isolated from a respiratory specimen obtained from an 18-year-old woman (case NY25) at a physician’s office in Ulster County, New York, in February 2015. The genome of this clinical isolate (15–4054; Table 1) had a close similarity to the vaccine strain CL68578, and the NYSDOH epidemiology team confirmed contact between this patient and an active member of the military. However, the mutations distinguishing the genome of this isolate from that of the vaccine strain exclude possible transmission from this source. Evolution of this p-like virus from the vaccine strain is highly likely, considering the ability of HAdV-4 to establish persistent infections in gut lymphoid tissue (*36*) and that vaccinated persons shed the infectious-nonattenuated vaccine strain in their stool (*37*).

 Our molecular epidemiology study of HAdV-4 infections in nonvaccinated military recruits in training during 1997–2011 demonstrated the lack of circulation of vaccine-like strains in the military environment during the 15 years of clear dominance of this re-emerging type as a causative agent of febrile respiratory illness in US recruit training camps (*16*). Unfortunately, no studies have reported genome typing data for civilian isolates obtained during the same period. A p-like variant designated 4p4 was identified as the only HAdV-4 genomic variant detected among military trainees at the US Coast Guard Training Center in Cape May through 2011 and, albeit with relatively low prevalence, as the only p-like variant circulating at the other 7 military training sites under surveillance (*16*) (A.E. Kajon, unpub. data).

Conceivably, exposure of the general population to the nonattenuated vaccine strain could have continued through fecal shedding from persons vaccinated during 1971–1997. Another possibility is that the p-like variants could have been circulating among civilian communities at low prevalence since the 1950s, when they were first identified (*2*). This topic warrants additional research and continued surveillance to further our understanding of the dynamics and routes of transmission of respiratory HAdVs that have the ability to establish persistent infection in the gut lymphoid tissue.

On the basis of the severity of the clinical presentation of some cases in this study, the HAdV-4 vaccine currently licensed for military use should be considered a potentially valuable resource to prevent disease in susceptible populations living in closed communities, such as college settings, summer camps, and long-term care facilities. Our data and reports of cases of severe ARD associated with HAdV-4 infection in Italy and Singapore (*38*,*39*) suggest that the role of this HAdV type in the etiology of adult civilian ARD might have been underestimated in the absence of access to molecular (or other) typing resources. Further, in view of the results of this study and previous research documenting the contribution of HAdV infection to influenza-negative ILI (*29*), the inclusion of HAdV in differential diagnostic test panels would be invaluable to better assess the role of HAdVs as causative agents of severe respiratory illness and to prevent unnecessary treatment of patients with influenza-negative ILI with anti-influenza agents. In addition, while the failure to detect influenza virus does not guarantee the virus was never present, the detection of HAdV in these cases assists in alleviating concerns regarding influenza vaccine failure. Finally, the potential differences in pathogenicity, transmissibility, and fitness between p-like and a-like genomic variants of HAdV-4 that would explain the marked predominance of a-like variants in the examined collections of HAdV-4–positive respiratory specimens representing sampling of ARD in civilian and military populations in the United States over the past 5 decades (*16*,*40*) deserve further investigation.
